# Motor Developmental Outcomes in Children Exposed to Maternal Diabetes during Pregnancy: A Systematic Review and Meta-Analysis

**DOI:** 10.3390/ijerph18041699

**Published:** 2021-02-10

**Authors:** Diana Arabiat, Mohammad AL Jabery, Vivien Kemp, Mark Jenkins, Lisa C. Whitehead, Gary Adams

**Affiliations:** 1 School of Nursing and Midwifery, Edith Cowan University, Perth 6027, Australia; v.kemp@ecu.edu.au (V.K.); m.jenkins@ecu.edu.au (M.J.); l.whitehead@ecu.edu.au (L.C.W.); 2 Maternal and Child Nursing Department, Faculty of Nursing, The University of Jordan, Amman 11942, Jordan; 3 Counselling and Special Education Department, Faculty of Educational Sciences, The University of Jordan, Amman 11942, Jordan; m.algabery@ju.edu.jo; 4 Queen’s Medical Centre, School of Health Sciences, The University of Nottingham, Nottingham NG7 2HA, UK; Gary.Adams@nottingham.ac.uk

**Keywords:** fine motor skills, gross motor skills, child development, psychomotor development, developmental delay, intra-uterine life

## Abstract

Studies on the association of maternal diabetes with motor development in children provide inconsistent findings. We searched MEDLINE/PubMed, EMBASE, Emcare, PsycINFO, and Google Scholar databases for primary observational, case–control, or cohort studies that report on the motor development of children exposed to maternal diabetes during pregnancy. Quality appraisal and data extraction were performed independently and in duplicate. A meta-analysis of summary measures was performed using random-effect models. Eighteen studies were identified for inclusion, however, only 13 were included in the meta-analysis. Exposure to maternal diabetes during pregnancy was associated with a lower pooled motor development in children and a decrease in both gross and fine motor development. Among all other factors, pre-existing diabetes and other gestational comorbidities, such as hypertension and obesity, or low socioeconomic status, also affect child development. Therefore, among children of diabetic mothers, those with other gestational comorbidities or pre-existing diabetes were more likely to be at risk developmentally.

## 1. Introduction

Children with typical development follow a pattern of developing gross and fine motor skills that allows them to know when they are developing well. The development of gross motor skills includes the assessment of muscle control, coordination, and locomotion, while the development of fine motor skills includes the control and coordination of body segments to achieve more complex movement and perceptual skills [1]. Delayed gross and fine motor development is usually noticed when the child fails to meet the normative development milestones by the normative age [2]. Poor motor development in infants and children may have long-term negative consequences for a child’s later development [3], and it is often indicative of more generalised developmental delays and disabilities in children [4].

It is a widely held view that motor development in children can be influenced by both genetics and environmental factors [5]. There are known predictors of motor development delay, such as low birth weight and premature birth [6], pregnancy complications [7], low maternal intelligence [8], and low education level [9]. There is also evidence that maternal diabetes can have deleterious effects on the developing foetus, as well conditions such as maternal hyperglycaemia, ketonaemia, and recurrent changes in glucose status [7,10]. Iron deficiency is also associated with diabetes [11] and can also adversely affect neurodevelopment in humans [12]. During pregnancy, a hyperglycaemic environment of the intrauterine life negatively impacts foetal neural development [13].

The association of maternal diabetes with motor development in child has been evaluated by several case–control [14,15] or cohort studies [13,16,17], with controversial conclusions. For example, in a recent study by Alamolhoda et al. [18] in Iran, findings showed that gestational diabetes (GDM) can be a powerful risk factor for motor developmental delay after adjusting for crucial variables. On the contrary, two retrospective longitudinal cohort studies based on smaller sample sizes from the United States [19,20] and Sweden [21] revealed that maternal diabetes was not related with the motor development of children.

With the advent of better perinatal care, many women are now closely monitored for blood glucose, and this has greatly reduced adverse outcomes for infants since the 1980s [22]. However, it is necessary to obtain more data to evaluate the association between maternal diabetes and motor developmental delay in children. It is suggested that motor developmental delays may be more subtle and harder to detect because results from studies are mixed and inconsistent [7,23], and the neuroplasticity of the developing brain means that remediation may be possible [24]. To address this knowledge gap, a systematic literature review and meta-analysis are needed to confirm whether exposure to maternal diabetes during intrauterine life is associated with delayed motor development in children.

Systematically synthesised information on the associations between intrauterine exposure to diabetes and motor development is lacking. This study will shed light on this topic and assist in identifying research gaps and provide scientific evidence regarding this association. This will help in identifying children at risk of developmental delay and would provide healthcare professionals with more information to deliver early diagnosis and interventions.

## 2. Materials and Methods

We performed a systematic review and meta-analysis in accordance with a published protocol (PROSPERO *registration number:* CRD42020182739). The Joanna Briggs Institute (JBI) methodology for systematic reviews and the Preferred Reporting Items for Systematic Review [25] and Meta-Analysis (PRISMA) guidelines [26] were used to report the findings.

### 2.1. Review Question and Eligibility

This review aimed to address the following question: Is there an association between exposure to maternal diabetes during pregnancy and motor development in children?

This review considered studies that report on children born to mothers with diabetes. Participants must be children of either gender, aged 12 years or less. The exposure of interest is maternal diabetes. More specifically, maternal diabetes includes diabetes in pregnancy, whether pre-existing or gestational. We included outcomes of motor development, such as fine motor and gross motor milestones. Only motor development outcomes measured by standardised tools were included. This review considered all analytical observational studies that have evaluated the impact of intrauterine foetal exposure to maternal diabetes, with no limitation as to the type of maternal diabetes. We excluded studies with unclear indicators of maternal diabetes during pregnancy, and studies published in languages other than English. There was no limitation on the date of publication.

### 2.2. Search Strategy and Study Selection

The initial search for this review was performed in duplicate using the search strategy outlined in the electronic Supplementary Material (Supplementary Text S1). The search was performed from June–September 2020. The search was carried out using keywords and medical subject heading terms (MeSH) that were modified for each database. MEDLINE/PubMed, Excerpta Medica dataBASE (EMBASE), Emcare, PsycINFO, and Google Scholar databases were searched using the identified index terms and search strategy. Following the search, identified citations were collated and uploaded into EndNote, and then duplicates were removed. The reference list of all included studies was screened for additional studies. Titles and abstracts were screened by two independent reviewers (V.K., M.J.) for assessment against the inclusion criteria for the review. Potentially relevant studies were retrieved in full, and their citation details were imported into the Joanna Briggs Institute System for the Unified Management, Assessment and Review of Information (JBI SUMARI) [25]. The selected full text citations were assessed in detail against the inclusion criteria by two independent reviewers (V.K., M.J.). Reasons for the exclusion of full text studies that did not meet the inclusion criteria were recorded and reported in the systematic review (Supplementary Table S1), leaving the other citations to be appraised. Any disagreements that arose between the reviewers at each stage of the study selection process were resolved through discussion with and inclusion of a third reviewer (M.A.).

### 2.3. Data Extraction and Assessment of Methodological Quality

Data were extracted from papers included in the review using the standardised data extraction tools in JBI SUMARI by two independent reviewers (Supplementary Tables S2 and S3). Each reviewer independently assessed the methodological quality of the included studies using standardised critical appraisal instruments from the JBI. All studies, regardless of the results of their methodological quality assessment, underwent data extraction and synthesis (where possible). Any discrepancy in quality assessment between reviewers was resolved through discussion.

### 2.4. Data Synthesis and Analysis

Data were pooled in a statistical meta-analysis using JBI SUMARI [25] and meta-analysis was performed using random-effect models. Summary measures of the effect size for standardised motor development outcomes were expressed as mean differences. Where means, standard deviations (SD), and effect estimates were not available, they were calculated from data where possible. Where SD data were not available, and standard error data were available, standard error values were converted into SD values. Means, SDs, and sample sizes for the group of children born to mothers with diabetes and those without diabetes were used to calculate effect size. An effect size of 0.2 was considered small, an effect size of 0.5 was considered moderate, and an effect size of 0.8 was considered large [27]. Statistical heterogeneity was quantified using I^2^ statistics. We were not able to perform stratified analysis by type of diabetes for the gross and fine motor scores given the lack of available data related to the type of diabetes or the limited number of studies.

## 3. Results

**Figure 1:**
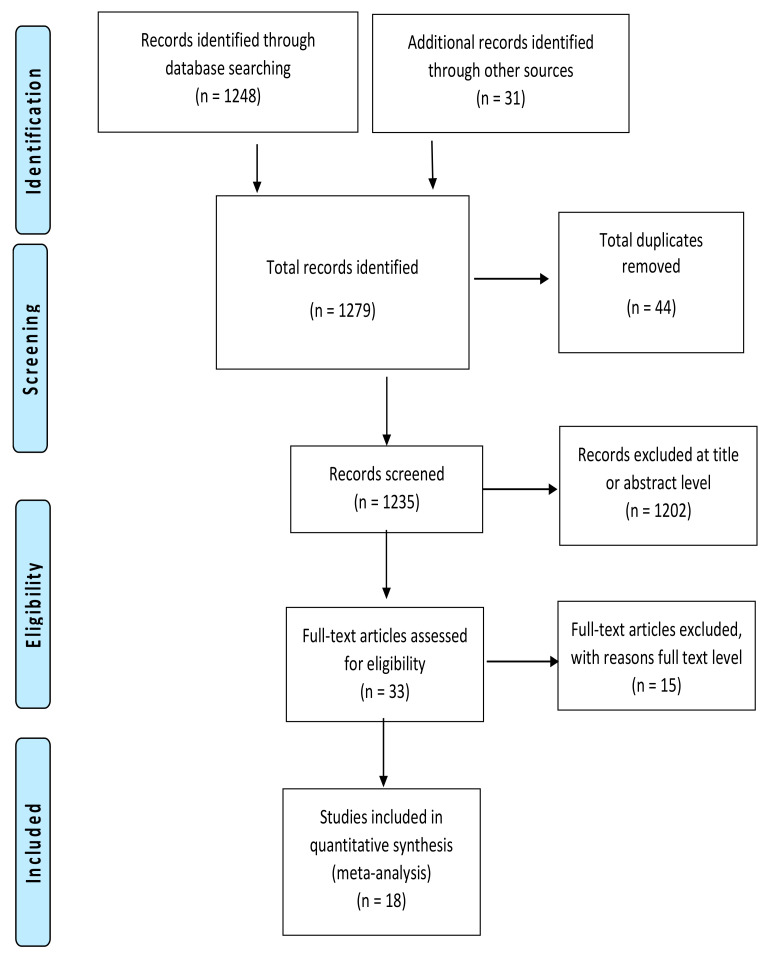
PRISMA flow diagram: Summary of studies search and selection process

The search strategy identified 1279 records, of which 44 duplicates were removed (Figure 1). There were 1203 records excluded at the title and abstract level, leaving 33 articles to be screened at the full text level. Of these, 15 were excluded with reasons, leaving 18 studies included in the review (Table 1).

### 3.1. Methodological Quality

Overall, the case–control studies were of a high quality (Supplementary Tables S4 and S5). Other than Biesenbach et al. [14], which scored positively on 8 out of 10 questions, all studies scored positively on all questions. For the cohort studies, excluding all non-applicable results, all other questions resulted in yes answers, except for three studies where there were 2 no answers for one study [28], and 1 no answer for two studies [29,30]

### 3.2. Characteristics of Included Studies

**Figure 2:**
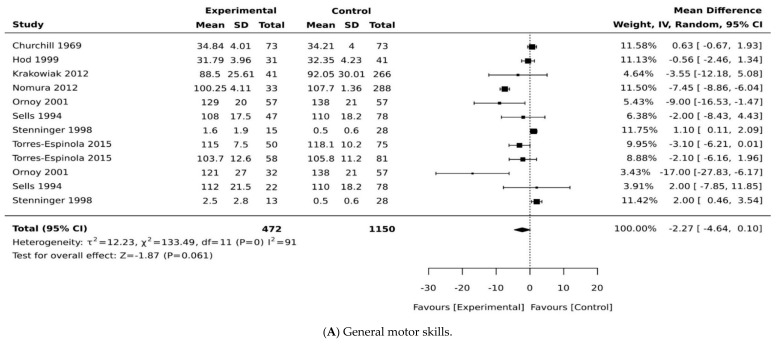

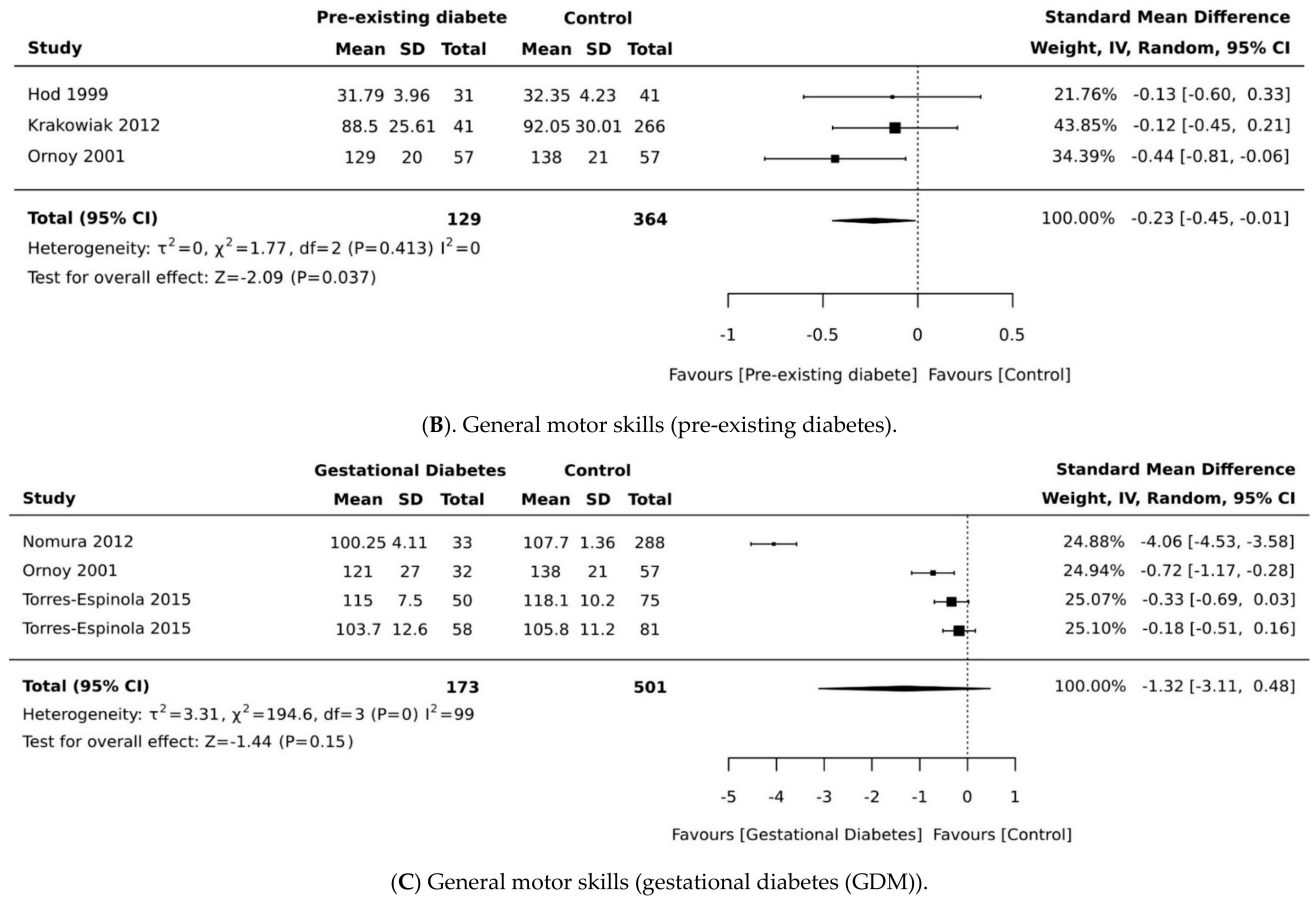
Effects of maternal pregnancy on general motor development. Forest plots comparing the differences in (**A**) motor development in all type of diabetes, (**B**) motor development between children born to mothers with pre-existing diabetes and no diabetes, and (**C**) forest plots comparing the differences in motor development between children born to mothers with and without gestational diabetes.

**Figure 3:**
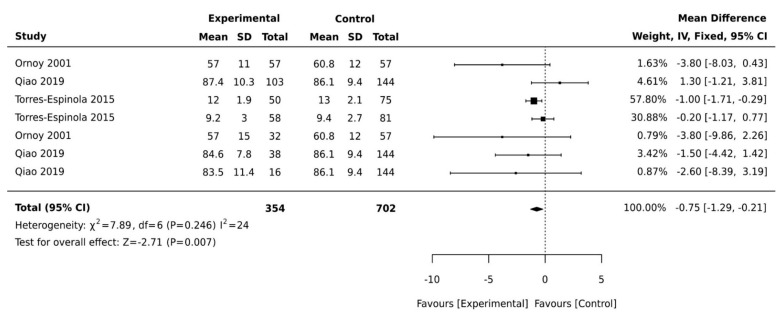
Forest plots comparing the differences in gross motor development between children born to mothers with and without maternal diabetes.

**Figure 4:**
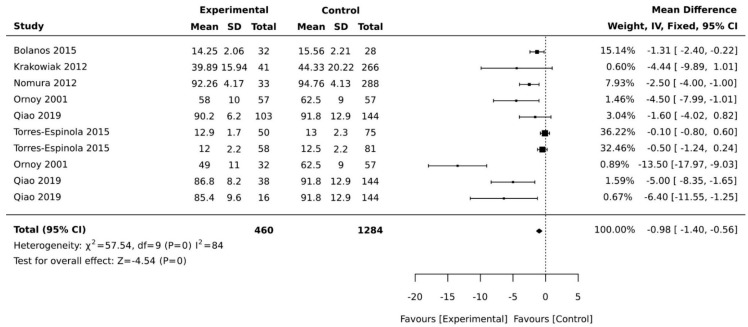
Forest plots comparing the differences in fine motor development between children born to mothers with and without maternal diabetes.

Of the 18 studies included, 10 were case–control studies, and 8 were cohort studies. Most studies came from high human development index countries, with the exception of Mexico, a medium development index country. In this review, four of the included studies used the same cohort [31,32,33,34] but reported on a different aspect of motor development; therefore, all were included in the review and only one was included in the meta-analysis (Table 2 and Table 3). Three studies used multiple regression analysis in reporting its findings and could not be included in our meta-analysis as the statistical coefficient of motor development cannot be reliably extracted from the regression equations (see Table 3). This resulted in the exclusion of these studies prior to our meta-analysis [16,17,35]. Outcome measures were heterogeneous across studies. We present the main characteristics of included studies in Table 1, Table 2 and Table 3. We subdivided motor development outcomes in this meta-analysis into three domains: general motor development, gross motor development, and fine motor development. The results of all effect sizes for the motor development outcomes are presented in forest plots in Figure 2, Figure 3 and Figure 4.

### 3.3. Exposure to Maternal Diabetes and Motor Development

We identified eight studies that examined the association of maternal diabetes with measures of motor development (Table 1). Only seven studies were pooled in a meta-analysis for general motor scores. Daraki et al. [17] reported adjustable data related to children born to mothers with and without diabetes, but these data could not be pooled because they were difficult to extract. In this study, the authors reported no association between glucose intolerance and obesity in early pregnancy and motor development in children. However, the study did not provide data for the matched comparison groups or a measure of error to compare motor scores among groups. One study [20] provided data for two cohorts, “early entry” and “late entry”, with no clear definition for the groups. In the meta-analysis, we pooled the standard deviation of the mean (SDM) using both cohorts. In another study [21], data were provided for children according to their experience of “hypoglycaemia” after birth, and both cohorts were included in the meta-analysis. Ornoy et al. [32] provided extractable data for two types of diabetes (pre-existing and gestational diabetes) and both groups were included in the main meta-analysis. In Torres-Espinola et al. [30], both cohorts of children assessed at 6 months and 12 months were included in the meta-analysis.

In the main meta-analysis including children born to mothers with pre-existing diabetes and GDM, the pooled weighted mean difference was −1.87 (95% CI, −4.64, 0.10; *p* = 0.061; I^2^ = 91%, Figure 2A), suggesting that children born to mothers with diabetes have significantly lower motor scores compared with control groups. We have performed a sensitivity analysis by including studies with only children born to mothers with pre-existing diabetes and found a pooled mean difference of −2.09 (95% CI, −0.45, −0.01; *p* = 0.037; I^2^ = 0%, Figure 2B), suggesting lower motor scores in children born to mothers with pre-existing diabetes. When the analysis was re-run, substituting mothers with pre-existing diabetes from the same Ornoy et al. cohort [32] with Ornoy et al.’s cohort of mothers with GDM [32] and other GDM groups [28,30], the pooled mean difference changed to −1.44 (95% CI, −1.32, 0.48; *p* = 0.15; I^2^ = 99%, Figure 2C). It should be noted that only two studies [21,30] found no significant differences in motor development between children born to mothers with and without diabetes.

### 3.4. Exposure to Maternal Diabetes and Gross Motor Development

Ten studies assessed gross motor development; however, only four could be pooled in the meta-analysis. Two studies used multiple regression data, which could not be pooled with the weighted mean differences extracted from the rest of the studies. Adane et al. [16] found that children born to mothers with diabetes had a higher risk of developmental delay, particularly gross motor skills, compared to the control group. This was consistent with Ghassabian et al. [35], who found that children born to mothers with GDM took longer to achieve major motor developmental milestones, such as sitting without support or walking.

Another two studies were not included as they assessed gross motor development by reporting the time the children started to walk [14] or the percentages of children with delayed gross motor development compared to children with typical development [13,14]. Biesenbach et al. [14] examined differences in the time children started to walk among two groups of children born to mothers with diabetes and nephropathy and those without nephropathy. There was no significant difference in the time children started to walk and these data could not be pooled as the control group was also diagnosed with diabetes and the study did not use a validated tool. Girchenko et al. [13] noted that overweight, obesity, and pre-eclampsia are also associated with motor developmental delay in children. The meta-analysis was performed using data from four studies. Ornoy et al. [32] included two cohorts of children born to mothers with pre-exiting diabetes and GDM, while Qiao et al. [29] included three different cohorts of children with neonatal hypoglycaemia and Torres-Espinola et al. [30] included cohorts of children aged 6 months and children aged 12 months. The pooled weighted mean difference was −2.71 (95% CI, −1.29, −0.21; *p* = 0.007; I^2^ = 24%, Figure 3), suggesting that children born to mothers with diabetes have significantly lower gross motor scores compared with control groups.

### 3.5. Exposure to Maternal Diabetes and Fine Motor Development

Ten studies reported on the association of maternal diabetes with fine motor development in children. Since Girchenko et al. [13] performed a stratified analysis by the mothers’ weight and presented data as percentages, this study could not be included in the meta-analysis. Stenninger et al. [21] used the manual dexterity subscale, which could not be pooled with other developmental scales used in the other studies. Stenninger et al. [21] found no significant differences in manual dexterity scores for children born to mothers with and without diabetes, however, children with hypoglycaemia showed lower motor scores compared to children without hypoglycaemia. The meta-analysis of six studies indicated a significant pooled mean difference of −4.57 (95% CI, −1.40, −0.56; *p* < 0.001; I^2^ = 84%, Figure 4).

## 4. Discussion

This meta-analysis found that children born to mothers with diabetes experience delayed motor development when compared to children born to mothers without diabetes, particularly for children born to mothers with pre-existing diabetes. There was also evidence of a low motor score in children born to mothers with GDM; however, there were insufficient studies and heterogeneity was too high to draw conclusions about these outcomes. Other gross and fine motor skills were also adversely affected among this population. We noted significantly lower pooled motor scores in children exposed to maternal diabetes compared with comparator groups with an overall effect of −2.71 motor points for gross motors skills and −4.54 motor points for fine motor skills.

It is worth noting that the pooled motor development score listed for motor development outcomes was unadjusted. Maternal obesity was a confounder in this meta-analysis as the majority of studies did not adjust findings by BMI, or other major confounders such as glycaemic control or gestational morbidities. Only one study adjusted data for weight gain during pregnancy [30], two studies adjusted data for birth weight and gestational age [15,19], and three studies adjusted for education level [12,20,28], thus, reported motor scores can be considered as independent of those confounders.

In this review, four studies matched in terms of some important confounders, such as age, socioeconomic status, and gestational age [30,31,32,33,34], yet residual confounding likely exists even after matching groups in those studies. This suggests that environmental influence on child motor development starts during intrauterine life and remains during growth.

Low socioeconomic alone or in combination with exposure to GDM increases the risk of neurobehavioral problems in children [28]. In addition, the influence of diabetes and other gestational comorbidities is also closely related to maternal overweight/obesity. For example, several studies reported that obesity is a strong predictor of maternal diabetes, including pre-existing diabetes and GDM [37,38]. Among all environmental factors, maternal pre-pregnancy overweight/obesity is the one that affects child motor development the most [13]. In a study performed in different diabetes cohorts with different means of BMI, children from diabetic and overweight mothers performed worse in the different motor development tests than those born to mothers with diabetes and normal weight [13]. However, when diabetes and control mothers came from a similar cohort, sharing the same parental obesity and glucose tolerance levels, the differences between their children’s neurodevelopment were not significant [17]. While those studies reported on the motor development of children exposed to maternal diabetes or not, they were not considered for this meta-analysis due to lack of standardisation among scores or our inability to reliably separate the statistical coefficient of motor development.

Our findings are congruent with earlier reports [13,28,39] that state that influences of other gestational comorbidities and environmental factors are closely related to a child’s development. Among all intrauterine factors, gestational comorbidities such as hypertension [13] and obesity [13,16,17,35] also affect child development and, therefore, among children of diabetic mothers, those with other gestational comorbidities were more likely to be at risk developmentally.

Another confounder in this review may be the type of maternal diabetes, which has been suggested previously to have a link with glycaemic control [40]. Five out of 18 studies did not differentiate the type of diabetes and considered all diabetic mothers as a single group [14,16,19,20,29]. When separated, our meta-analysis showed that children from mothers with pre-existing diabetes had worse motor development than those from GDM mothers. This may relate to the fact that GDM often involves a less severe form of hyperglycaemia of shorter duration. We noted that two studies were limited to women with pre-existing diabetes and had the largest difference in motor scores between groups [12,32], whereas studies limited to GDM [28,30] had the lowest difference between groups. In the study by Torres-Espinola et al. [30], children born to diabetic mothers had the lowest motor skill at 18 months, but this difference was lost in adjusted models.

## 5. Limitations

This study has several limitations. First, there are the small number of studies included in each analysis and the significant heterogeneity between studies calculated using I^2^ statistics. We noted significant heterogeneity for both general motor and fine motor skills that can be related to remarkable differences between studies in the type of measures used, time of assessment, and type of maternal diabetes. Therefore, our findings should be interpreted cautiously. Second, including only studies published in the English language raises a limitation by possibly missing out on studies published in other languages [41]. Third, the greatest limitation concerns the limited number of studies adjusted for important confounders, such as glycaemic control and other comorbidities. Some studies included in this review were minimally adjusted or unadjusted for important confounders and, therefore, the findings must be interpreted carefully. While some of the studies were adjusted to perinatal factors such as gestational age, birthweight, and neonatal hypoglycaemia, it remains unclear what the roles of other gestational comorbidities, such as maternal obesity, gestational hypertension, glycaemic control, and low socioeconomic status, are and to what degree those factors may have contributed to our findings.

## 6. Conclusions

Maternal diabetes during pregnancy was associated with reduced motor development in children. This study underscores the importance of the type of diabetes, particularly glycaemic levels, in reducing the disparities in motor development among children born to mothers with diabetes. For children born to mothers with diabetes, interventions should focus on maintaining glycaemic control and optimum weight during pregnancy in order to ensure children’s improved motor development. It is penitent also for policy makers to consider these variables when thinking about strategies to cope with the increased complexity and new challenges posed by the number of children appears to have developmental delay [42].

## Figures and Tables

**Table 1:** Characteristics of included studies.

Reference	Country	Study Design	Age at Assessment	Diabetes Group	N	Comparator Group	N	Developmental Outcome
Krakowiak et al. [12]	USA	Case–control	2–6 years	Pre-existing and GDM	45	No diabetes	235	Motor scores
						No diabetes, but had Autism Spectrum Disorder (ASD)	266	
Girchenko et al. [13]	Finland	Case–control	Mean age 3.5 years	GDM and normal weight	85	No diabetes and normal weight	1652	Gross motor
				Type 1 diabetes and normal weight	4	No diabetes and normal weight	383	
				GDM and overweight	69	No diabetes and overweight	212	
				Type 1 diabetes and overweight	4	No diabetes and obese		
				GDM and obese	94			
				Type 1 diabetes and obese	1			
Biesenbach et al. [14]	Austria	Cohort study	3 years	Diabetes with stage IV nephropathy	10	Diabetes without nephropathy	30	Gross motor
Hod et al. [15]	Israel	Cohort study	1 year	Type 1 diabetes	21	No diabetes	41	Motor scores
				Type 2 diabetes	10			
Adane et al. [16]	Australia	Case–control	0–6 years	Diabetes during pregnancy	61	No diabetes	710	Gross and fine motor
Daraki et al. [17]	Greece	Case–control	4 years	GDM and normal weight	35	No diabetes and normal weight	463	Motor scores
				GDM and overweight	7	No diabetes and overweight	166	
				GDM and obese	14	No diabetes and obese	87	
Churchill et al. [19]	USA	Cohort study	6 months–1 year	Class A diabetes and no acetonuria	73	No diabetes	73	Motor scores
				Class A diabetes and acetonuria	55	No diabetes	55	
Sells et al. [20]	USA	Cohort study	6 months–2 years	Early entry at age 6 months	51	No diabetes at child age 6 months	83	Motor scores
				Early entry at age 12 months	62	No diabetes at child age 12 months	83	
				Early entry at age 24 months	47	No diabetes at child age 24 months	78	
				Late entry at age 6 months	32			
				Late entry at age 12 months	31			
				Late entry at age 24 months	22			
Stenninger et al. [21]	Sweden	Cohort study	6–12 years	Hypoglycaemic	13	No diabetes	28	Motor scores
				Non-hypoglycaemic	15			
Nomura et al. [28]	USA	Case study	2–6 years	GDM	214	No diabetes	191	Fine motor
Qiao et al. [29]	China	Case–control	2 years	Group 1 hypoglycaemic <2 h after birth	103	No diabetes	144	Gross and fine motor
				Group 2 hypoglycaemic 2–24 h after birth	38			
				Group 3 hypoglycaemic >24 h after birth	16			
Torres-Espinola et al. [30]	Spain	Case–control	6–18 months	GDM at child age 6 months	58	Normal weight at child age 6 months	81	Gross motor
				GDM at child age 18 months	50	Normal weight at child age 18 months	75	
						Overweight at child age 6 months	44	
						Overweight at child age 18 months	43	
						Obese at child age 6 months	32	
						Obese at child age 18 months	29	
Ornoy et al. [31]	Israel	Cohort study	Mean age 8 years	Pregestational diabetes	57	No diabetes	57	Gross and fine motor
Ornoy et al. [32]	Israel	Cohort study	Early school age	Pre-existing diabetes	57	No diabetes	57	Gross and fine motor
				GDM	32			
Ornoy et al. [33]	Israel	Cohort study	5–12 years (young vs. old)	GDM	32	No diabetes	57	Gross and fine motor
Ratzon et al. [34]	Israel	Cohort study	Mean age 8 years	Pre-existing diabetes	57	No diabetes	57	Gross and fine motor
Ghassabian et al. [35]	USA	Case–control	4 months–2 years	GDM	N/A	Gestational comorbidities (hypertension–eclampsia)	4897	Gross motor
Bolaños et al. [36]	Mexico	Cohort study	Mean age 8 years	GDM	32	No diabetes	28	Fine motor

GDM: Gestational diabetes.

**Table 2:** Summary of results of included studies.

Reference	Measurement	Adjusted Analysis	Summary of Main Results	Included in Meta-Analysis
Krakowiak et al. [12]	Mullen Scales of Early Learning (MSEL)	Yes	Diabetes may be associated with neurodevelopmental problems in offspring. Proportionately more mothers of children in the ASD and developmental delay groups had either type 2 diabetes or GDM. The risk having a child with developmental delay relative to type 2 diabetes was significantly increased among obese women.	Yes
Girchenko et al. [13]	Ages and Stages Questionnaire (ASQ)	Yes	Maternal early pregnancy overweight, obesity, and pre-eclampsia are independently associated with neurodevelopmental delay in children. GDM increased the odds of developmental delay but can be partially explained by maternal overweight/obesity and other disorders.	No
Biesenbach et al. [14]	Month of life when starting to walk	No	Both groups of children born to mothers with and without diabetes started to walk at the same age.	Yes
Hod et al. [15]	Bayley Scales of Infant Development (MDI) and Psychomotor Developmental Index (PDI)	No	Both MDI and PDI scores were significantly lower in infants of diabetes mothers compared with the control group. Moreover, infants of mothers with type 2 diabetes had lower scores on the PDI and motor quality index.	Yes
Adane et al. [16]	Ages and Stages Questionnaire (ASQ)	Yes	Children born to chronically obese women were more likely to be at risk developmentally. Children of mothers with diabetes during pregnancy were at slightly greater risk of developmental delay, particularly gross motor skills, compared to women without diabetes in pregnancy.	No
Daraki et al. [17]	The McCarthy Scales of Children’s Abilities (MSCA)	Yes	Obesity, maternal glucose tolerance in early pregnancy, and GDM were not associated with child neurodevelopment, including motor development.	No
Churchill et al. [19]	Bayley Scales of Infant Development (BSID); neurological posturing scales	No	The infants of diabetic mothers differed significantly from matched controls in Bayley mental and motor scores at 6 months and in posturing rating scale at 1 year. Infants of mothers who had diabetes and were acetonuria positive showed significantly greater developmental deficits than matched controls. Infants born to mothers who had diabetes and were acetone negative did not differ from their matched controls. The presence or absence of acetonuria, not the severity of diabetes, explained the differences in child development.	Yes
Sells et al. [20]	Bayley Scales of Infant Development (BSID)	Yes	No significant differences between groups with relation to motor development.	Yes
Stenninger et al. [21]	Screening for Minimal Brain Dysfunction (MBD); Assessment Battery (Movement ABC); Griffith’s Mental Developmental Test (GMDT eye and hand coordination scale)	No	No differences in neurological examination, but children in the neonatal hypoglycaemia group had significantly higher scores in the minimal brain dysfunction test. No significant differences in the Movement Assessment Battery Test (MABT).	Yes
Nomura et al. [28]	Developmental Neuropsychological Assessment (NEPSY)	Yes	Children exposed to both GDM and low socioeconomic status (SES) showed compromised neurobehavioural outcomes; the risk of ADHD was synergistically associated with exposure to both GDM and low SES.	Yes
Qiao et al. [29]	Gesell developmental test (GDT, Chinese revised version)	No	There was no difference reported in gross or fine motor skill acquisition or adaptability between the controls and any infant in Group A.	Yes
Torres-Espinola et al. [30]	Bayley Scales of Infant Development (BSID)	Yes	Although not significant, at age 18 months, gross motor scores were lower in the overweight, obese, and GDM groups compared to the control group. Motor skill at 18-month adjusted analysis of infants of GDM mothers had lower scores, but this disappeared in adjusted models.	Yes
Ornoy et al. [31]	Bruininks–Oseretsky Motor Development Test (BOMDT);	No	Children born to mothers with diabetes had significantly lower scores on the Bruininks–Oseretsky Motor Development Test (BOMDT); children born to diabetic mothers had more soft neurological signs and lower gross and fine motor movement achievements than children born to non-diabetic mothers.	Yes
Ornoy et al. [32]	Bruininks–Oseretsky Motor Development Test (BOMDT)	No	Children whose mothers did not have diabetes scored significantly higher on the BOMDT than children of mothers with any type of diabetes. However, differences between children of diabetic mothers and control group children lessened over time.	Yes
Ornoy et al. [33]	The Touwen–Prechtl neurological examination; Bender Visual Gestalt Test for the evaluation of eye–hand coordination; Goodenough Draw a Man test; Bruininks–Oseretsky Motor Development Test (BOMDT)	No, but matched by age, birth order, and socioeconomic status.	Younger children in the index group had significantly lower scores on the BOMDT, but this difference was not present in the older index group children. There were no differences between groups on the Touwen–Prechtl neurological examination. Overall, even though children born to mothers with GDM had higher rates of lower fine and gross motor skill scores in the younger age group, when compared with control group children, these differences diminished with age.	Yes
Ratzon et al. [34]	Bruininks–Oseretsky Test of Motor Proficiency (BOTMP)		Children born to mothers with diabetes had more fine and gross motor difficulties than children born to mothers without diabetes. There was a negative correlation between a mother’s high HbA1C and high acetonuria and the children’s BOTMP scores. Environmental variables and gross motor development positively correlated only for children born to mothers with diabetes.	Yes
Ghassabian et al. [35]	World Health Organisation (WHO) major milestones/time to achieve motor milestones	Yes	Children of mothers with diabetes or GDM took longer to achieve major motor milestones measured—sitting without support, walking with assistance, and walking alone—than children of mothers without diabetes or GDM, independent of maternal obesity. Children of mothers with hypertensive diseases also took longer to achieve milestones, but this difference disappeared after adjustment for perinatal factors.	No
Bolaños et al. [36]	Purdue Pegboard Dexterity Test (PPDT)	No	The GDM group children had significantly lower scores in graphic and spatial abilities. Motor skills were significantly lower and there were more soft neurological signs in children whose mothers had GDM than children in the control group.	No

GDM: Gestational diabetes; ASD: Autism spectrum disorder; ADHD: Attention deficit/hyperactivity disorder.

**Table 3:** Summary of studies on motor development included in this review.

Reference	Primary Developmental Outcome	Main Findings Presented (Mean (SD), Percentage, Adjusted Model)	
Krakowiak et al. [12]	Fine motor	DM: 38.98 (28.57), Ctrl: 44.32 (0.60)	*p* = 0.01 *, effect size (−0.21)
	Fine motor	DM and ASD: 27.11 (20.94), Ctrl: 27.79 (17.75),	*p* = NS, effect size (−0.04)
	Standard motor	DM: 86.15 (41.70), Ctrl: 92.06 (30.57)	*p* = 0.04 *, effect size (−0.16)
	Standard motor	DM and ASD: 75.00 (32.48), Ctrl: 74.35 (27.86),	*p* = NS, effect size (0.02)
Girchenko et al. [13]	Fine motor	GDM: 21 (8.5%) with DD vs. 17 (6.9%) with normal development	
	Gross motor	GDM: 15 (6.1%) with DD vs. 21 (8.5%) with normal development	
	Fine motor	Type 1 DM: 3 (33.3%) with DD vs. none with normal development	
	Gross motor	Type 1 DM: 2 (22.2%) with DD vs. none with normal development	
	Adjusted model for children born to mothers with GDM vs. no diabetic disorders (mild DD)		
	Fine motor	0.83 (0.51–1.36)	*p* = NS
	Gross motor	0.85 (0.48 = 1.51)	*p* = NS
	Adjusted model for children born to mothers with GDM vs. no diabetic disorders (severe DD).		
	Fine motor	1.11 (0.63–1.95)	*p* = NS
	Gross motor	1.40 (0.83–2.35)	*p* = NS
Biesenbach et al. [14]	Gross motor	Mean age child started to walk: Nephropathy: 11 months (2), without nephropathy 11 months (1)	*p* < 0.05 *, effect size (0.00)
Hod et al. [15]	Motor quality	PGDM: 31.79 (3.96), Ctrl: 32.35 (4.23)	*p* = NS, effect size (−0.14)
Adane et al. [16]	Gross and fine motor skills	Adjusted RR (95% CI): 1.22 (0.67, 2.24)	
Daraki et al. [17]	Motor scale scores	GDM: Adjusted β-coefficient (95% CI): −1.50 (−5,80, 2.79).	
Churchill et al. [19]		Class A diabetes and acetonuria: 31.50 (4.26), Ctrl: 33.23 (4.29),	*p* = 0.01 *, effect size (−0.40).
		Class A diabetes and no acetonuria: 31.50 (4.26), Ctrl: 33.23 (4.29),	*p* = NS, effect size (0.16)
Sells et al. [20]	Bayley Motor Scale 6 months	Early entry: 109 (16.2), Ctr: 108 (15)	*p* = NS, effect size (0.06).
	Bayley Motor Scale 6 months	Late entry: 104 (17.0), Ctrl: 108 (15)	*p* = NS, effect size (−0.25)
	Bayley Motor Scale 12 months	Early entry: 104 (15.5). Ctrl: 103 (15.8)	*p* = NS, effects size (0.06)
	Bayley Motor Scale 12 months	Late entry: 102 (17.2), Ctrl: 103 (15.8)	*p* = NS, effect size (−0.06).
	Bayley Motor Scale 24 months	Early entry: 108 (17.5), Ctrl: 110 (18.2)	*p* = NS, effect size (−0.11)
	Bayley Motor Scale 24 months	Late entry: 112 (21.5), Ctrl: 110 (18.2)	*p* = NS, effect size (0.10)
	Bayley Motor Scale 6 months	Group with major malformation: 89 (19.0), Ctrl: 109 (14.8)	*p* < 0.0001 **, effect size (−1.17)
	Bayley Motor Scale 12 months	Group with major malformation: 93 (21.0), Ctrl: 106 (14.3)	*p* < 0.024 *, effect size (−0.72)
Stenninger et al. [21]	MBD scores	Hypoglycaemic: 2.5 (2.8), Ctrl: 0.5 (0.6)	*p* < 0.05 *, effect size (0.99)
	MBD scores	Non-hypoglycaemic: 1.6 (1.9), Ctrl: 0.5 (0.6)	*p* < 0.05 *, effect size (0.78)
	Manual dexterity (fine motor)	Hypoglycaemic: 1.96 (2.4), Ctrl: 0.93 (1)	*p* = NS, effect size (0.56)
	Manual dexterity (fine motor)	Non-hypoglycaemic: 1.33 (1.7), Ctrl: 0.93 (1)	*p* = NS, effect size (0.29).
	Balls skills (gross motor)	Hypoglycaemic: 2.12 (2), Ctrl: 1.75 (1.9)	*p* = NS, effect size (0.19)
	Balls skills (gross motor)	Non-hypoglycaemic: 2.13 (2.2), Ctrl: 1.75 (1.9),	*p* = NS, effect size (0.18)
	Static and dynamic balance	Hypoglycaemic: 1.5 (2.4), Ctrl: 0.59 (1.2)	*p* = NS, effect size (0.48)
	Static and dynamic balance	Non-hypoglycaemic: 0.83 (1.3), Ctrl: 0.59 (1.2)	*p* = NS, (0.19)
	Total ABC scores	Hypoglycaemic: 5.58 (6), Ctrl: 3.27 (2.4)	*p* = NS, effect size (0.51)
	Total ABC scores	Non-hypoglycaemic: 4.3 (3.9), Ctrl: 3.27 (2.4),	*p* = NS, effect size (0.32)
	GMDT eye and hand coordination	Hypoglycaemic: 5.9 (2.4), Ctrl: 6.6 (1.6)	*p* = NS, effect size (−0.34)
	GMDT eye and hand coordination	Non-hypoglycaemic 7.2 (1.7), Ctrl: 6.6 (1.6)	*p* = NS, effect size (0.36)
Nomura et al. [28]	Sensorimotor	GDM: 92.26 (4.17), Ctrl: 94.76 (4.13)	*p* = NS, effect size (−0.60)
Qiao et al. [29]	Gross motor	Group 1: 87.4 (10.3), Ctrl: 86.1 (9.4)	*p* = NS, effect size (0.13)
	Fine motor	Group 1: 90.2 (6.2), Ctrl: 91.8 (12.9)	*p* = NS, effect size (−0.16)
	Gross motor	Group 2: 84.6 (7.8), Ctrl: 86.1 (9.4)	*p* = NS, effect size (−0.17)
	Fine motor	Group 2: 86.8 (8.2), Ctrl: 91.8 (12.9)	*p* = NS, effect size (−0.46)
	Gross motor	Group 3: 83.5 (11.4), Ctrl: 86.1 (9.4)	*p* = NS, effect size (−0.25)
	Fine motor	Group 3: 85.4 (9.6), Ctrl: 91.8 (12.9)	*p* = NS, effect size (−0.56)
Torres-Espinola et al. [30]	Composite motor	GDM 6 months: 103.7 (12.6), Ctrl: 105.8 (11.2)	*p* = NS, effect size (−0.18)
	Fine motor	GDM 6 months: 12 (2.2), Ctrl: 12.5 (2.2)	*p* = NS, effect size (−0.25)
	Gross motor	GDM 6 months: 9.2 (3.0), Ctrl: 9.4 (2.7)	*p* = NS, effect size (−0.07)
	Composite motor	GDM 18 months: 115 (7.5), Ctrl: 118.1 (10.2)	*p* = 0.09 **, effect size (−35)
	Fine motor	GDM 18 months: 12.9 (1.7), Ctrl: 13 (2.3)	*p* = NS, effect size (−0.05)
	Gross motor	GDM 18 months: 12 (1.9), Ctrl: 13 (2.1)	*p* = 0.041 *, effect size (−0.50)
Ornoy et al. [31]	Bruininks total	(Mean, SE): DM: 129.2 (3.9), Ctrl: 138.2 (3.7)	MD = −9.0, *p* = 0.008.
	Bruininks gross motor	DM: 57.2 (1.7). Ctrl: 60.8 (1.7)	MD = −3.6, *p* = 0.03
	Bruininks fine motor	DM: 58.0 (1.9), Ctrl: 62.5 (1.7),	MD = −4.5, *p* = 0.01.
Ornoy et al. [32]	Bruininks total	Diabetic mothers: 129 (20), Ctrl: 138 (21)	*p* < 0.05 *, effect size (−0.44)
	Bruininks total	GDM: 121 (27), Ctr: 138 (21)	*p* < 0.05 *, effect size (−0.70)
	Bruininks gross motor	Diabetic mothers: 57 (11), Ctrl: 60.8 (12)	*p* < 0.05 *, effect size (−0.33)
	Bruininks gross motor	GDM: 57 (15), Ctrl: 60.8 (12)	*p* < 0.05 *, effect size (−0.28)
	Bruininks fine motor	Diabetic mothers: 58 (10), Ctrl: 62.5 (9)	*p* < 0.05 *, effect size (−0.47)
	Bruininks fine motor	GDM: 49 (11), Ctrl: 62.5 (9)	*p* < 0.05 *, effect size (−1.34)
Ornoy et al. [33]	Bruininks total	GDM (young): 113 (28), Ctrl: 128(23)	*p* < 0.05 *, effect size (−0.59)
	Bruininks total	GDM (old): 131 (26), Ctrl: 127 (18)	*p* = NS, effect size (0.18)
	Bruininks gross motor	GDM (young): 52.1 (15.5), Ctrl: 59.2 (130)	*p* < 0.05 *, effect size (−0.08)
	Bruininks gross motor	GDM (old): 61.8 (14.7), Ctr: 66.8 (10.3)	*p* = NS, effect size (−0.39)
	Bruininks fine motor	GDM (young): Bruininks 45.9 (11.6), Ctr: 53.4 (9.7)	*p* < 0.05 *, effect size (−0.70)
	Fine motor	GDM (old): 45.9 (11.6), Ctrl: 53.4 (9.7)	*p* < 0.05 *, effect size (−0.70).
Ratzon et al. [34]	Total motor scores	DM: 129.2 (29.2), Ctrl: 138.2 (27.6)	*p* = 0.008 **, effect size (−0.32)
	Gross motor	DM: 57.2 (12.7), Ctrl: 60.8 (13.2)	*p* = 0.03 *, effect size (−0.28)
	Fine motor	DM: 58.0 (14.6), Ctrl: 62.5 (12.7)	*p* = 0.01 *, effect size (−0.33)
Ghassabian et al. [35]	Time to achieve gross motor milestone	GDM up to age 24 months: Adjusted HR (95% CI): 0.84 (0.75–0.93)	*p* = 0.002 **
Bolaños et al. [36]	Right hand	GDM: 9.47 (1.46), Ctrl: 10.18 (1.77)	*p* = NS, effect size −0.44.
	Left hand	GDM: 9.31 (1.3), Ctrl: 9.61 (1.83)	*p* = NS, effect size (−0.19)
	Both hands	GDM: 14.25 (2.06), Ctrl: 15.56 (2.21)	*p* = 0.023 *, effect size (−0.061)

* *p* < 0.05; ** *p* < 0.001; NS: Non-significant; DM: Diabetes mellitus; GDM: Gestational diabetes; PGDM: Pregestational diabetes; Ctr: Control; DD: Developmental delay; SD: Standard deviation; SE: Standard error; MD: Mean difference.

## Data Availability

The data presented in this review are available on request from the corresponding author.

## Supplementary Materials

The following are available online at https://www.mdpi.com/1660-4601/18/4/1699/s1, Text S1: Search strategy. Table S1: Excluded studies at full text; Table S2: Characteristics of case control included studies; Table S3: Characteristics of included cohort studies; Table S4: Case control study critical appraisal results; Table S5: Cohort study critical appraisal results.
