# Supplementary material for: Motor Developmental Outcomes in Children Exposed to Maternal Diabetes during Pregnancy: A Systematic Review and Meta-Analysis
Source: Int J Environ Res Public Health. 2021 Feb 10;18(4):1699. doi: 10.3390/ijerph18041699 (PMC7916519; doi:10.3390/ijerph18041699)
Supplement: Supplementary file 1 [file ijerph-18-01699-s001.pdf]

Supplementary Material

Supplementary Text S1. Search strategy

Search strategy

(((Child[Title/Abstract] OR Children[Title/Abstract] OR Offspring[Title/Abstract] OR Infants of diabetic mothers[Title/Abstract] OR Offspring of diabetic mothers[Title/Abstract])) AND (pregnancy in diabetes OR diabetes in pregnancy OR antepartum diabetes OR gestational diabetes OR diabetic mothers)) AND (motor development OR perceptual motor development OR childhood development OR early childhood development OR psychomotor development OR motor coordination OR motor skills OR gross motor development OR fine motor development)

Supplementary Table S1. Excluded studies at full text

Study	Exclusion reason
de Moura et al., 2010	Aggregated, not itemised developmental scores.
Hinkle et al., 2012	No mention of diabetes.
Kimmerle et al., 1995	Results are descriptively reported.
Kowalczyk et al., 2002	Results are not reported in means and SD’s. There is one aggregate score for whole the scale, which means the contribution of motor skills is unknown.
Petersen et al., 1988	Results are reported in percentages, not means and SD’s
Rizzo et al., 1991	Motor skill development is not specifically mentioned.
Rizzo et al., 1995	Correlations between maternal diabetes severity and child development only. No comparison with age-controlled peers.
Rizzo et al., 1997	Children’s scores are correlated with maternal diabetes severity. No discrete motor skills scores.
Rizzo et al., 1994	No specific motor skills scores.
Silverman et al., 1991	No specific motor skills scores.
Torabi et al., 2012	Results do not specifically state the relationship between maternal gestational diabetes and motor skills development. They only state general developmental delay.
Yamashita et al., 1996	Results do not report on motor skills.
Yeung et al., 2017	No specific reporting on the effect of maternal diabetes

Supplementary Table S2. Characteristics of case control included studies

Study	Country	Setting/context	Participant characteristics	Group A description and sample	Group B description and sample	Exposures/variables measured	Description of main results
Biesenbach G. 2000.	Austria	Prospective study comparing development of children of diabetic mothers, with and without Stage IV diabetic nephropathy born between 1985-1993	N =40 Women with diabetes and their children contacted between 3 -7 years post-partum	n= 30 children from the pregnancies of 28 diabetic women without nephropathy	n =10 children born to 10 diabetic women with Stage IV nephropathy	At 3 years of age, body weight, height, month of life starting to walk, month of like starting to talk, number of infectious diseases per child.	At age 3, children of the mothers with nephropathy were more likely to be below the 50th percentile in height and weight, both groups started to walk at the same age. Children of mothers with nephropathy began speaking on average three months later than the other children.
Churchill JA. 1969.	United States of America	To determine whether the neurological and psychological status of children born to diabetic mothers differs from that of children born to non-diabetic mothers	Participants were drawn from the Perinatal Study of the National Institute of Neurological Diseases and Blindness, and included only children from pregnancies resulting in singleton births who had also	Group 1 N= 134 (67 matched pairs, 34 (50.7%) F, and 33 (49.3%) M) diabetic mothers and diabetic mothers with Class A diabetes and acetonuria.	Group 3 n= 110 (55 matched pairs, 26 (47.2%) F, 29 (52.8%) M non-diabetic mothers and Class 2 diabetic mothers with acetonuria; Group 4 n= 36 (18 matched pairs, 6 (33.3) F, 12 (66%) M nondiabetic mothers and	Duration of pregnancy; birth weight, Bayley scales, Neurological posturing scales, Stanford-Binet IQ test at 4 years of age	The infants of diabetic mothers differed significantly from matched controls in Bayley mental and motor scores at 6 months, posturing rating scale at 12 months and Binet IQ at 4 years. Infants of mothers who were diabetic and acetonuria positive showed significantly greater developmental deficits

			been administered the Bayley mental and motor examination at 8 months of age or the Stanford -Binet IQ test at 4 years of age. N= 237	Group 2 n= 146 (73, matched pairs 30 (41%) F, 43(59%) M non - diabetic and diabetic mothers with Class A diabetes but no acetonuria	Class 2 diabetic mothers without acetonuria; Group 5 n= 24 matched pairs non diabetic mothers and mothers with all classes of diabetes not tested fro acetonuria		than matched controls. Infants born to mothers who were diabetic and acetone-negative diabetic did not differ from their matched controls. The authors conclude that it was the presence or absence of acetonuria, not the severity of diabetes that explained the differences found.
Bolaños L. 2015.	Mexico	To determine whether child born to mothers with gestational diabetes show neuropsychological developmental delays at age 7 years, born between March 1998 and September 1999. 215 children	N= 215 (excluded were unschooled children, twins, children with neurological disorders secondary to diseases acquired postnatally). Final sample n= 64, Children were all similar in terms of age, gender and handedness	Control group, no maternal diabetes n= 28, mean age 8.82 (0.593) years, 16 (57%) F, 12 (43%) M	Gestational diabetes group n= 32, mean age 8.88 (0.575) years, 17 (53%) F, 15 (47%) M	Birth weight in Kg, number of weeks gestation, mother's age at birth; The Child Neurological Evaluation, Purdue Pegboard Dexterity Test, and Wechsler Intelligence Scale for Children	The gestational diabetes group children had significantly lower scores on graphic, spatial abilities and working memory index of the WISC-IV. Bianual skills, were significantly lower and there were more soft neurological signs in children whose mothers had gestational diabetes than children in the control group.

Hod M. 1999.	Israel	Longitudinal study of mothers and infants	N= 72 pregnant women at 20-28 weeks gestation	n= 41 infants of matched control mothers with no diabetes	n= 31 Infants born to mothers with pregestational diabetes (21 type 1, 10 type 2)	Psychomotor development in infants at one year of age using the Bayley Scales of Infant Development (MDI and PDI)	Both MDI and PDI scores were significantly lower in infants of diabetic mothers compared with the control group. Infants of diabetic mothers were less alert and responsive and more likely to be fretful and cry. Moreover,infants of mothers with type 2 diabetes had lower scores on the PDI and motor quality index.
Levy-Shiff R. 2002.	Israel	Longitudinal case control study of maternal adjustment offspring outcomes of high risk pregnancies	N= 153 women without diabetes, or pregestational, gestational diabetes and and their singleton offspring, evaluated at 1 year of age	N= 53 Women with PGDM, Group 2 N =51 GDM Mean age 32.6 (13.2SD) years	n= 49 non diabetic pregnanciesmean age 31.8 (5.0 SD) years	Pregnant women: Cognitive appraisal of pregnancy as a challenge and threat (Folkman & Lazarus, 1985); Ways of coping checklist (Folkman & Lazarus, 1985), Social Support Questionnaire (Crnic, Greenberg, & Slough, 1986), Pregnancy-related emotions (Folkman & Lazarus, 1985), The revised Beck	Women with diabetes during pregnancy were more likely to experience negative emotions and negative cognition about the pregnancy than women without diabetes. Infants of mothers with PGDM and GDM scored significantly lower than infants of non-diabetic mothers on the MDI of the Bayley Scales. Infants of of pPGDM scored

						Depression Inventory (Beck & Steer, 1987), State–Trait Anxiety Inventory (Spielberger, Gorsuch, & Lushene, 1973), Burnout Questionnaire (Pines & Aronson, 1981) and Symptom Checklist (Lips, 1985). Infants at one year: Bayley Scales of Infant Development—Second Edition (BSID-II; Bayley, 1993).	significnatly lower on the PDI than infants of mothers GDM and nondiabetic mothers. Infants of PGDM and GDM mothers displayed more negative and fewer positive behaviours than infants of mothers without diabetes.
Ornoy A. 1998.	Israel	Longitudinal study of offspring of mothers with and withhout diabetes born beteen 1982-1987	N = 171 children born to mothers with and without diabetes,	Control group children born to mothers without diabetes n= 57, mean age 8.26± 1.78 years (range 5.5-12.1), 25 (44%) F, 32 (56%) M	n= 57 children born to mothers with type 1 or type 2 diabetes mean age 8.09±1.77 years, range 5.2-12.1 years) 28 (49%) F, 29 (51) %M	The Touwen–Prechtl neurological examination for minor nervous dysfunction; The Pollack tapper test; theWechsler Intelligence Scales for Children, Revised (WISC-R, 1974); Bender Visual Gestalt test; Bruininks-Oseretsky Motor Development	There were no differences between groups on the WISC-R. Children born to diabetic mothers had significantly lower scores on the Bruininks-Oseretsky Motor Development test; Children born to diabetic mothers had more soft neurological signs and lower gross and fine motor

						test; Southern California Integration Test, and The Conners Abbreviated Parent–Teacher Questionnaire	movement achievements that children born to non-diabetic mothers.
Ornoy A. 1999.	Israel	Longitudinal study of offspring of mothers with PGDM, GDM, and without diabetes	N= 89 Children born to mothers with PGDM, GDM, and without diabetes between 1982-1987 ranging from 5.2–12.1 years.	Control group, no maternal diabetes n= 57; mean age 8.3 (SD 1.7)26 (46%) F, 31 (56%) M. The group was further divided into Younger children (n= 31, 55% 5-8 years) and Older children (n= 26, 45% 9-12 years)	N= 32 children born to mothers with gestational diabetes mean age 8.5 (SD 2.1) years, 13 (41%) F, 19 (59%)M. The group was further divided into Younger children (n= 15, 47% 5-8 years and Older children (n= 17, 53%) 9-12 years)	The Touwen–Prechtl neurological examination, Wechsler Intelligence Scales for Children Revised (WISC-R, 1974); Bender Visual Gestalt test26 for the evaluation of eye–hand coordination; Goodenough Draw a Man test; Bruininks–Oseretsky Motor Development test; Southern California Integration Test; Conners Abbreviated Parents–Teachers' Questionnaire; The Pollack tapper test; Achenbach's questionnaire for the measurement of behaviour and Home observation for measurement of	Younger children in the index group had significantly lower scores on the Bruininks–Oseretsky Motor Development test, but this difference was not present in the older index group children. there were no differences between groups on the Touwen–Prechtl neurological examination. Overall even though children born the mothers with gestational diabetes had higher rates of attention deficits, lower cognitive scores, and lower fine and gross motor skill scores in the younger age group, when compared with control group children, these

						environment questionnaire. Data analysis further divided the children into a younger group (ages 5-8 years and older group (ages 9-12 years	differences diminished with age.
Ornoy A. 2001.	Israel	Longitudinal study of early school age offspring of mothers with and without diabetes, born between 1982-1987	N = 114 early school age children	Children born to mothers without diabetes n= 57 25 (44%) F, 32 (56 %) M	Children born to mothers with type 1 or Type 2 diabetes n= 57, 28 (49%) F; 29 (51%) M. Group 3 Children born to mothers with gestational diabetes n=32 13 (41%) F, 19 (59%) M	The Touwen & Prechtl neurological examination; The Pollack Taper Test; Revised Wechsler Intelligence Scales for Children (WISC-R, 1974), Bender Visual Gestalt Test, Bruininks-Oseretsky Motor Development Test, Southern California Integration Test, The Conners abbreviated Parent-Teacher's Questionnaire	Children whose mothers had no diabetes scored significantly higher on the Bruininks-Oseretsky Motor Development Test than children of mother with any type of diabetes. However differences between offspring of diabetic mothers and control group children lessened over time
Ratzon N. 2000.	Israel	Longitudinal study examining the motor development of children born to mothers wirth and without diabetes	N= 114 children born to mothers between 1982-1987with and without diabetes	Control goup n = 57; 25 (44%) F, 32 (66%) M; mean age 8.29	Chidren of mothers with type 1 and type 2 diabetes n= 57, 28 (49%) F, 29 (51%) M; mean	Bruininks-Oseretsky Test of Motor Proficiency (BOTMP). Home Observation for Measurement of the Environment	Children born to mothers with diabetes had more fine and gross motor difficulties than children born to mothers without diabetes. Negative

			matched for age, SES, parental education and profession, birth order and family size	(1.78SD) years.	age 8.09 (1.77SD) years.	(HOME) for school children Maternal state of diabetes control	correlation between a mother's high HbA!C and high acetonuria and the children's BOTMP scores<br>Environmental variables and gross motor development positively correlated only for children of diabetic mothers
Sells CJ. 1994.	United States of America	Theee year longitudinal Diabetes in Early Pregnancy study of neurological development of infants born to insulin dependent diabetic and non-diabetic mothers	N= 250 (18 infants lived out of state, 22 mothers declined to participate, 11 lost to follow up) leaving sample size of 199<br>Among diabetic mothers, those with more education were more like to remain in the study.	Control group infants of non-diabetic mothers n =90	Infants of diabetic mothers n = 109. Early entry mothers (enrolled for study within 21 days of conception) n = 70, Late entry mothers (enrolled after 22 days of conception) n =39	Bayley Scales of Infant Development at 6, 12 and 36 months of age Vineland Adaptive Behavior Scales at 6, 12, and 36 months of age Stanford- Binet Intelligence Scale (fourth edition) at 36 months of age Peabody Picture Vocabulary Test, Form Mat 36 months of age Mean Length of Utterance for morphemes at 36 months of age	Cognitive development - no significant differences between groups Motor development - no significant differences between groups Language development - significant differences between groups on two measures of verbal development at 3 years that persisted after adjustment for parental education Growth characteristics -infants of diabetic mothers weighted significantly less at birth than non diabetic mothers Infant malformations - at 3

							years, nearly 15% if infants of diabetic mothers had major malformations compared to 7.8% of infants of non-diabetic mothers
Stenninger E. 1998.	Sweden	Longitudinal study of infants born to diabetic mothers from 1986-1988	N = 58 infants, of whom 30 were chosen for follow up from a birth cohort of 76 infants of diabetic mothers based on preprandial capillary blood glucose levels.	Control group n=28 children of non diabetic mothers, mean age 7.8 (0.4SD) years, 14 (50%) F, 14 (50%) M	Post natal hypoglycasemia (blood glucose level < 1.5mmol/l) n = 13 Mean age 7.9 (0.7SD) years, 9 (69%) F, 4 (31%) M ; Without postnatal hypoglycaemia (blood glucose level > 1.5mmol/l), mean age 7.7 (0.6SD) years, 10 (66%) F, 5 (33%) M	General and neurological examination including screening for minimal brain dysfunction Movement Assessment Battery for Children aged 6 to 12 years (Movement ABC) Griffiths' mental developmental scales (2–8 years) EEG recording (Walter Graphtek GmbH, PL-EEG, version 2.1)	No differences in mothers of infants in perinatal risk factors. No significant differences in measurements of height, weight and head circumference. No differences in neurological examination, but children in the neonatal hypoglycemia group had significantly higher scores in the minimal brain dysfunction test. No significant differences in the Movement Assessment Battery test No significant differences in the EEG tests

Supplementary Table S3. Characteristics of included cohort studies

Study	Country	Setting/context	Participant characteristics	Groups	Outcomes measured	Description of main results
Daraki V. 2017.	Greece	Prospective Rhea mother–child cohort study February 2007 - February 2008	N= 875 children underwent neuro-developmenal assessment from October 2011 to January 2013 (26 children with pervasive developmental disorders and 11 mothers with missing data were excluded from analysis) leaving 772mother-child pairs, 378 (49.9) F, 394 (51.1%) M	group 1 n=691 Women with gestational diabetesGroup 2 n =452 women with fasting gloucose and insluin serum meansurements in early pregnancyGroup 3 n =378 mother-child pairs with data from maternal IQ	Parental overweight/obesity; maternal glucose intolerance in early pregnancy and GMD; Neuropshyological assessment of offspring at 4 years of age	Maternal obesity associated with significant score reduction of offspring general cognitive ability, perceptual performance, quantitative ability and executive functions at age 4. Maternal obesity associated with increased behavioural problems and ADHD symptoms at age 4; Paternal obesity, maternal glucose tolerance in early pregnancy and GDM was not associated with child neurodevelopment.
Ghassabian A. 2016.	United States of America	Upstate KIDS, population-based cohort examining the relationship between infertility treatment and child	N =4909; 2368 (48.2%) F; 2551 (51.8%) M 1142 (29.4%) conceived through infertility treatment. 2368	Group 1 Sitting without support n = 4893Group 2 Standing with assistance n= 4892Group 3 Hands- and -knees crawling n= 4897Group 4 Walking with assistance n =	Time to achieve major motor milestones	Children of mothers with diabetes or GDM took longer to achieve major motor milestones measured - sitting without support, walking with assistance and walking alone than children of mothers without diabetes

		development, 2008-2010	(48.2) F, 2541 (51.8%) M	4897Group 5 Standing alone n= 4897Group 6 Walking alone n =48976		or GDM, independent of maternal obesity. Children of mothers with hypertensive diseases also took longer to achieve milestones, but this difference disappeared after adjustment for perinatal factors.
He XJ. 2020.	China	Prospective cohort study of mother-child pairs May 2014 - May 2017	N= 783 early pregnant women recruited, 228 excluded from analysis (151 left study before birth, 65 excluded before 1 year examination, 12 incomplete examination) n =555 mother child pairs.	Control group no maternal diabetes n =378, 201 (53.2) F, 177 (46.8%) MGDM group n = 177, 92 (52%) F, 85 (48%) M	Maternal height, weight, fatty acid analysis, Infant Measures of neurodevelopment : Bayley Scales of Infant Development (Chinese version), Mental and Psychomotor Developmental Index	Control group infants scored significantly higher on both PDI and MDI than infants whose mothers had GDM. Maternal age and saturated fatty acids were independently related to with infant neurodeveopment at one year.
Girchenko P. 2018.	Finland	Prediction and Prevention of Pre-eclampsia and Intrauterine Growth Restriction (PREDO) Study of live born children between 2006-2010.	N = 2504 mother -children dyads who enrolled when pregnant between 2006-2010 and were followed up between 2011-2012.	Group 1 Normal weight n= 1741 Normal weight mothers (1652 no diabetes, 85 GDM, 4 Type 1 diabetes), child's mean age in months at follow up 41.8 (8.2SD); 866 (49.7%) F, 875 (50.7%) M Overweight mothers	Ages and Stages Questionnaire (ASQ) measuring developmental milestones	Maternal early pregnancy overweight, obesity, and pre-eclampsia are independently associated with neurodevelopmental delay in offspring. Gestational diabetes increased the odds of developmental delay but can be partially explained by maternal overweight/obesity

				n = 456 (383 no diabetes, 69 gestational diabetes, 4 type 1 diabetes), child's mean age in months at follow up 42.3 (8.2SD), 231 (50.7) F, 225 (49.3%) M Obese mothers n = 307 (212 no diabetes, 94 GDM, 1 type 1 Diabetes), child's mean age in months at follow up 43.6 (8.4SD), 140 (45.6%) F, 167 (54.4%)M		and other disorders * the study may have contained milder cases of GDM due to increased screening for it in Finland*
Krakowiak P. 2012.	United States of America	CHARGE (Childhood Autism Risks from Genetics and the Environment) study, 2003 - 2010	N = 1004 Children aged between 2-6 years	Group 1 Children with austism spectrum Disorders n= 517, mean age at study enrolment 3.65 years (0.80SD), 81 (15.6), 436 (85.8) M<br>Group 2 Children with developmental delays n= 172 mean age at study enrollment 3.79 (0.76SD), 51 (42%)F, 121 (68%) MControl group children n=315 Mean age at study enrollment 3.54	Maternal metabolic conditions Autism Diagnostic Interview, Revised (ADI-R) The Social Communication Questionnaire Mullen Scales of Early Learning (MSEL) and Vineland Adaptive Behavior Scales	Maternal metabolic conditions may be associated with neuro- developmental problems in offspring. Proportionately more mothers of children in the ASD and DD groups had either type 2 Diabetes or GDM. The risk having an offspring with ASD or DD relative to Type 2 diabetes was significantly increased among obese women

				(0.80SD), 59 (18.7%) F, 256 (81.3%) M		
Nomura Y. 2012.	United States of America	Longitudinal cohort study investigating the risk of ADHD in the offspring of mothers with GDM and low SES	N= 212 Children, mean age 4.1 years; 56(26.5%) F, 156 (73.5%) M	GDM absent n= 191; 52 (27.2%) F, 139 (72.8%) MGDM present n =21 4 (19%) F, 17 (81%) M	ADHD RS–IV34, Developmental Neuropsychological Assessment (NEPSY), The Wechsler Preschool and Primary Scale of Intelligence– Third Edition (WPPSI-III), Temperament Assessment Battery for Children Revised (TABC-R) at ages 3-4 years, and Behavior Assessment System for Children–2 (BASC-2) at 6 years	Both GDM and low SES alone and in combination increase the risk of ADHD, GDM and family SES influences the risk for ADHD; Offspring exposed to both GDM and low SES showed compromised neurobehavioural outcomes; and the risk of ADHD was synergistically associated with exposure to both GDM and low SES
Qiao LX. 2019.	China	Propective longitudinal study of offsdpring of diabetic mothers born with a low blood glucose level	N= 301 (382 enrolled in study, 81 infants lost to follow up), 139 (46%)F; 162 (54%) M tested at corrected age 2 years ± 2 months	Control group n= 144 infants born to mothers without diabetes, no neonatal hypoglycaemiasub group A1 n=103 neonates hypoglycemic <2 hours after birthsub group A2 n = 38 neonates hypoglycemic 2 - 24 hours after birthsub group Q3 n =16 neonates	Neurodevelopment as measured by Gesell developmental test (Chinese revised version)at age 2 years	The longer neonatal hypoclycaemia continued, the more adaptability was impaired, in infants in sub groups A2 and A3. But there was no difference reported in gross or fine motor skill acquisition, adaptability, language or social skills between the controls and any infant in Group A.

				hypoglycemic >24 hours after birth		
Adane AA.	Australia	Subset of Australian Longitudinal Study on Women's Health (ALSWH) 1973–78 cohort and Mothers and their Children's Health (MatCH) study.	N=771 of mothers and children who were of eligible age for the Ages and Stages Questionnaire (ASQ) and 708 children form the Australian Early Development Census (AEDC). All tested between 0 -66 months of age, details of sex not provided.	Developmental delay staus on ASQ n=771 Developmental vulnerability status on AEDC domains n =708	Gross motor skills; Gross and fine motor skills, Language and cognitive skills, Communication and general skills	Children born to chronically obese women were more likely to to be at risk developmentally. Children of mothers with diabetes during pregnancy were at slightly greater risk of developmental delay, particularly gross motor, language and cognitive skills compared to women without diabetes in pregnancy. No significant difference between diabetes in pregnancy and childhood physical and cognitive development.
Torres-Espinola FJ. 2015.	Spain	Case -control study of mother-child pairs recruited between 2007-2012	N= 331 health pregnant women with singleton pregnancies aged between 18-45 years, recruited between 12-34 weeks of pregnancy. due to attrition, sample size of n =215 at 6	Normal weight mothers n= 81 at 6 months 41 (50.6%) F, 40 (49.4%) M. At child age 18 months n =75Overweight mothers n = 44 at 6 months 24 (54.5%) F, 20 (45.5%) M. At child age 18 months n = 43Obese mothers n= 32 at 6 months. 15 (46.9	Bayley Scales of Infant Development, Third Edition (BSID-III) at age 6 and 18 months.	Although not significant, at age 18 months gross motor scores were lower in the overweight, obese and GDM groups compared to control group. Language was significantly lower at age 6 months for infants of obese mothers compared to controls, and the trend was lower but not significant for infants overweight and

			months and n =197 at child age 18 months	%) F, 17 (53.1%) M. At child age 18 months n =29GDM mothers n = 58 at 6 months. 26 (44.8%) F 32 (55.2%) M. At child age 18 months n =50		GDM mothers. At 18 months none of the significant differences remained for language. Motor skill at 18 months adjusted analysis of infants of GDM mothers had lower scores, but this disappeared in adjusted models.

Supplementary Table S4. Case control study critical appraisal results.

**Citation**	**Q1**	**Q2**	**Q3**	**Q4**	**Q5**	**Q6**	**Q7**	**Q8**	**Q9**	**Q10**
Biesenbach et al., 2000	Y	Y	Y	Y	Y	N	N	Y	Y	Y
Bolaños et al., 2015	Y	Y	Y	Y	Y	Y	Y	Y	Y	Y
Churchill et al., 1969	Y	Y	Y	Y	Y	Y	Y	Y	Y	Y
Hod et al., 1999	Y	Y	Y	Y	Y	Y	Y	Y	Y	Y
Ornoy et al., 1998	Y	Y	Y	Y	Y	Y	Y	Y	Y	Y
Ornoy et al., 2001	Y	Y	Y	Y	Y	Y	Y	Y	Y	Y
Ornoy et al., 1999	Y	Y	Y	Y	Y	Y	Y	Y	Y	Y
Ratzon et al., 2000	Y	Y	Y	Y	Y	Y	Y	Y	Y	Y
Sells et al., 1994	Y	Y	Y	Y	Y	Y	Y	Y	Y	Y
Stenninger et al., 1998	Y	Y	Y	Y	Y	Y	Y	Y	Y	Y
Yes percentage	100.0	100.0	100.0	100.0	100.0	90.9	90.9	100.0	100.0	100.0

Supplementary Table S5. Cohort study critical appraisal results.

**Citation**	**Q1**	**Q2**	**Q3**	**Q4**	**Q5**	**Q6**	**Q7**	**Q8**	**Q9**	**Q10**	**Q11**
Adane et al., 2018	Y	Y	Y	Y	Y	N/A	Y	Y	Y	N/A	Y
Daraki et al., 2017	Y	Y	Y	Y	Y	N/A	Y	Y	Y	N/A	Y
Ghassabian et al., 2016	Y	Y	Y	Y	Y	N/A	Y	Y	Y	N/A	Y
Girchenko et al., 2018	Y	Y	Y	Y	Y	N/A	Y	Y	Y	N/A	Y
Krakowiak et al., 2012	Y	Y	Y	Y	Y	N/A	Y	N/A	N/A	N/A	Y
Nomura et al., 2012	Y	Y	Y	Y	Y	N/A	Y	Y	N	N	Y
Qiao et al., 2019	Y	Y	Y	N	N/A	N/A	Y	Y	Y	N/A	Y
Torres-Espinola et al., 2015	Y	Y	Y	Y	Y	N/A	Y	Y	Y	N	Y
Yes percentage	100.0	100.0	100.0	88.88	88.88	0.0	100.0	88.88	77.77	0.0	100.0
